# Identification and validation of supervariants reveal novel loci associated with human white matter microstructure

**DOI:** 10.1101/gr.277905.123

**Published:** 2024-01

**Authors:** Shiying Wang, Ting Li, Bingxin Zhao, Wei Dai, Yisha Yao, Cai Li, Tengfei Li, Hongtu Zhu, Heping Zhang

**Affiliations:** 1 Department of Biostatistics, Yale School of Public Health, New Haven, Connecticut 06510, USA;; 2 Department of Applied Mathematics, The Hong Kong Polytechnic University, Hong Kong, China;; 3 Department of Statistics and Data Science, University of Pennsylvania, Philadelphia, Pennsylvania 19104-1686, USA;; 4 Department of Biostatistics, St. Jude Children's Research Hospital, Memphis, Tennessee 38105, USA;; 5 Department of Radiology, University of North Carolina at Chapel Hill, Chapel Hill, North Carolina 27599, USA;; 6 Biomedical Research Imaging Center, School of Medicine, University of North Carolina at Chapel Hill, Chapel Hill, North Carolina 27514, USA;; 7 Department of Biostatistics, University of North Carolina at Chapel Hill, Chapel Hill, North Carolina 27599, USA

## Abstract

As an essential part of the central nervous system, white matter coordinates communications between different brain regions and is related to a wide range of neurodegenerative and neuropsychiatric disorders. Previous genome-wide association studies (GWASs) have uncovered loci associated with white matter microstructure. However, GWASs suffer from limited reproducibility and difficulties in detecting multi-single-nucleotide polymorphism (multi-SNP) and epistatic effects. In this study, we adopt the concept of supervariants, a combination of alleles in multiple loci, to account for potential multi-SNP effects. We perform supervariant identification and validation to identify loci associated with 22 white matter fractional anisotropy phenotypes derived from diffusion tensor imaging. To increase reproducibility, we use United Kingdom (UK) Biobank White British (n = 30,842) data for discovery and internal validation, and UK Biobank White but non-British (n = 1927) data, Europeans from the Adolescent Brain Cognitive Development study (n = 4399) data, and Europeans from the Human Connectome Project (n = 319) data for external validation. We identify 23 novel loci on the discovery set that have not been reported in the previous GWASs on white matter microstructure. Among them, three supervariants on genomic regions 5q35.1, 8p21.2, and 19q13.32 have *P*-values lower than 0.05 in the meta-analysis of the three independent validation data sets. These supervariants contain genetic variants located in genes that have been related to brain structures, cognitive functions, and neuropsychiatric diseases. Our findings provide a better understanding of the genetic architecture underlying white matter microstructure.

White matter, as an essential part of the central nervous system, composes roughly half of the human brain (Filley and Fields 2016). White matter mainly consists of bundles of myelinated axons, or tracts, which connect various gray matter areas and coordinate communications among brain regions (Hagmann et al. 2008; Schmahmann et al. 2008). Functioning as a modulator of the distributed neural network, white matter is dynamically involved in learning and information processing (Fields 2008). The abnormal structure and dysfunction of white matter are related to a wide range of neurodegenerative and neuropsychiatric disorders, such as Alzheimer's disease (Gold et al. 2012; Lee et al. 2016), schizophrenia (Flynn et al. 2003; Cetin-Karayumak et al. 2020), and major depression disorder (Zou et al. 2008). The genetic analyses of white matter help elucidate biological mechanisms underlying learning and information processing and further deepen our understanding of the etiology of those brain-related diseases.

Diffusion tensor imaging (DTI) is a magnetic resonance imaging modality that enables the measurement of white matter microstructure in vivo (Le Bihan et al. 2001). Fractional anisotropy (FA) derived from DTI at each voxel is a simple and robust metric to quantify white matter integrity (Pfefferbaum et al. 2000). Moreover, white matter tracts extracted from DTI form a complex network of structural connections and shape communication and connectivity patterns. In general, white matter tracts with higher FA values have higher white matter integrity (Pfefferbaum et al. 2000). Evidence indicates that changes in FA values are associated with various neuropsychiatric disorders (Podwalski et al. 2021) and cognitive functions (Grieve et al. 2007). FA values are also highly heritable. The heritability of tract-averaged FA is estimated to range from 53% to 90% in a twin study (Kochunov et al. 2015) and from 31% to 66% based on SNPs (Zhao et al. 2021a). Therefore, FA values are useful for studying the genetic influence on white matter microstructure.

GWASs have been performed to study the genetic basis of white matter microstructure (Elliott et al. 2018; Rutten-Jacobs et al. 2018; Zhao et al. 2021a,b; Smith et al. 2021). For instance, Zhao et al. (2021a) performed the largest GWASs for DTI-derived phenotypes, including FA, mean diffusivity (MD), axial diffusivity (AD), radial diffusivity (RD), and mode of anisotropy (MA) along 21 white matter tracts. Genetic loci associated with tract-averaged FA have been identified. However, GWASs focus on the marginal effects of individual SNPs on phenotypes and suffer from limited reproducibility and difficulties in detecting multi-SNP and epistatic effects (Wu et al. 2010). Such multi-SNP and epistatic effects might account for additional heritability that cannot be explained by genetic variants identified in the GWASs.

As an alternative strategy, SNP-set analysis groups SNPs based on genomic regions or functional features and then tests their joint effects. Within the framework of SNP-set analysis, we consider the concept of supervariants. Similar to the concept of the gene, a supervariant is a combination of alleles in multiple loci. However, unlike a gene that is a physically connected region on a chromosome, the loci contributing to a supervariant can be anywhere in the genome (Song and Zhang 2014; Hu et al. 2020, 2021; Li et al. 2021). Supervariants adaptively aggregate signals of multiple alleles and are expected to account for complex multi-SNP effects even when they are located remotely. Previous genome-wide studies have shown the validity of supervariants and successfully identified supervariants and corresponding genetic variants for breast cancer (Hu et al. 2020), brain connectivity (Li et al. 2021), and COVID-19 related mortality (Hu et al. 2021; Liu et al. 2023).

In this study, we perform supervariant identification and validation to identify loci associated with 22 white matter FA phenotypes using a ranking and aggregation method (Song and Zhang 2014; Hu et al. 2020). To increase the reproducibility of results, we consider both internal and external validation. We use participants with White British ancestry from the UK Biobank (UKB) data set (*n* = 30,842) for supervariant identification and internal validation. The identified supervariants are further replicated in three external validation data sets with European ancestry: UKB White but non-British (UKBW; *n* = 1927), Europeans from the Adolescent Brain Cognitive Development study (ABCD; *n* = 4399), and Europeans from the Human Connectome Project (HCP; *n* = 319). For identified supervariants and selected SNPs, we perform biological annotation, gene-level analysis, and association lookups on the NHGRI-EBI GWAS catalog (Buniello et al. 2019). By performing supervariant identification and validation, we aim to detect novel and replicable loci associated with white matter FA phenotypes, which potentially improve our understanding of the genetic architecture of white matter microstructure.

## Results

### Discovery and internal validation of supervariants associated with white matter microstructure

**Figure 1.**
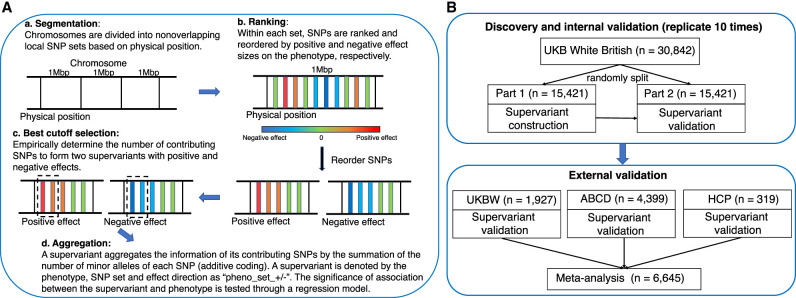
Supervariant identification and validation procedure. (*A*) Workflow of supervariant construction following a local ranking and aggregation procedure. (*B*) Workflow of discovery, internal validation, and external validation of supervariants. UKB White British data set is randomly split into two parts. On the first part, supervariants are constructed following the four steps in *A*. On the second part, the association between constructed supervariants and phenotype is validated. This discovery and internal validation procedure is repeated 10 times. Supervariants that can be discovered and validated multiple times on the UKB White British data set are regarded as reproducible supervariants. They are further validated on three external data sets with European ancestry. Then, meta-analysis is performed to combine the results. (UKB) UK Biobank, (UKBW) UKB White but non-British, (ABCD) Adolescent Brain Cognitive Development study, and (HCP) Human Connectome Project.

We perform supervariant identification and internal validation for 22 white matter FA phenotypes (mean FA of 21 white matter tracts and average FA across all the tracts) derived from the data set of the UKB White British (*n* = 30,842). The supervariant construction procedure follows a local ranking and aggregation method (Fig. 1A). It adaptively ranks and selects SNPs to form supervariants for a specific phenotype. First, we divide the whole genome into 2723 nonoverlapping local SNP sets and construct two supervariants, one with the positive effect and the other with the negative effect, for each predefined SNP set (Song and Zhang 2014; Hu et al. 2020). A supervariant denoted as “pheno_set_+/−” is the aggregation of selected SNPs within the SNP set with a positive or negative effect on the phenotype. A total of 2723 × 2 = 5446 supervariants are considered for each phenotype.

Our analysis considers the following discovery and internal validation procedure (Hu et al. 2021) shown in Figure 1B. The complete set is randomly divided into two sets with equal sizes (*n* = 15,421 for each set), one for the construction of supervariants and the other for validation. We apply the aforementioned ranking and aggregation method for supervariant construction on the first part of the data set. Then, after the construction of the supervariants, we validate the associations between constructed supervariants and white matter phenotype through linear regressions on the second part. We control for age (at imaging), sex, image site, age-squared, age and sex interaction, age-squared and sex interaction, and the top 10 principal components (PCs) in the regression to remove potential bias. We use 4.17 × 10^−7^ (i.e., 0.05/(2723 × 2 × 22)) as the threshold for the supervariant candidacy on the first part of the data set because 5446 supervariants and 22 phenotypes are considered. A supervariant is regarded as validated if its linear regression coefficient achieves the level of 0.05/22 significance on the second part of the data set. On the second part of the data set, we only adjust for the number of phenotypes instead of using the most stringent threshold that also adjusts for the number of selected supervariants, aiming to include more potential associations for further analysis. As compensation, we repeat the above procedure 10 times and retain the validated supervariants and their contributing SNPs to ascertain the reproducibility of the associations. Typically, genetic association analyses do not include internal validation, but we replicate our procedure 10 times as a safeguard strategy for detecting potential and stable signals. We provide empirical support for our strategy by performing simulation analyses to show that this procedure can control false positives (Supplemental Note S1).

**Figure 2.**
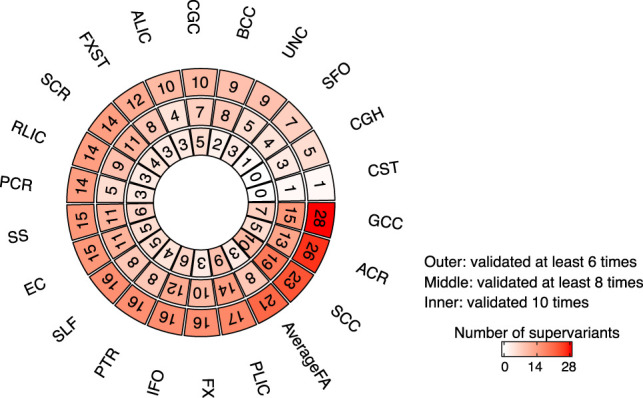
The number of discovered and validated supervariants in UKB White British (n = 30,842) with different times of replication. The *outer* layer counts the number of supervariants for each white matter FA phenotype that can be discovered and validated in at least six times replication, the *middle* layer counts the ones that can be discovered and validated in at least eight times, and the *inner* layer counts those can be discovered and validated in 10 times. The full names of 21 white matter tracts are detailed in Figure 3B.

At the significance level mentioned above, supervariants are discovered and validated multiple times across 10 times replication. We identify 90 supervariants in 10 times replication, 194 supervariants in at least eight times, and 314 supervariants in at least six times. The number of supervariants for each phenotype is shown in Figure 2. We focus on 314 supervariants that can be discovered and validated at least six times (Supplemental Table S1). According to the binomial distribution, the probability of a supervariant being validated in at least six out of 10 times replication by chance is 2.87 × 10^−14^ if the *P*-value on the second part of the data set is assumed to be uniformly distributed.

**Figure 3.**
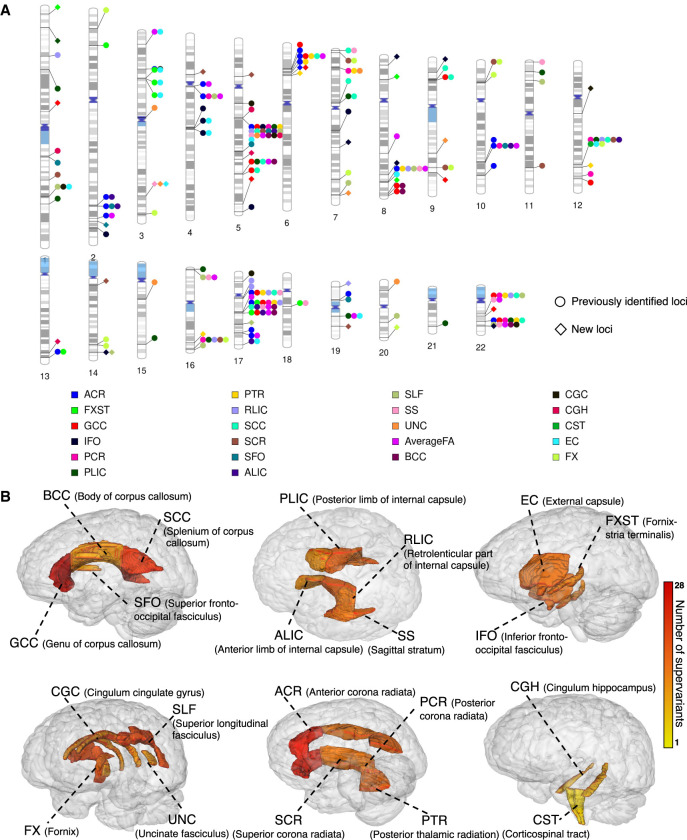
Identified supervariants associated with 22 white matter FA phenotypes in UKB White British (n = 30,842). (*A*) Ideogram of genomic regions influencing white matter FA phenotypes, including 78 previously identified regions and 23 additional regions identified in the current study. The colors represent the 21 white matter tracts (and the global average). Each signal point indicates that this white matter tract is associated with the genomic region. (*B*) The number of supervariants associated with 21 white matter tracts in human brain. The color scale represents the number of supervariants ranging from one to 28 associated with this white matter tract.

The physical locations of the identified supervariants on the chromosomes cover 123 SNP sets (Fig. 3A). Each SNP set corresponds to a genomic region on chromosomes. In Figure 3A, we observed that several genomic regions are linked to multiple white matter tracts. For instance, within the SNP set Chr5_83, 33 supervariants are identified, which are associated with mean FA of 18 white matter tracts and average FA, and within the SNP set Chr22_39, 18 supervariants are involved in the association with mean FA of 10 tracts and average FA. Among 123 SNP sets, 56 are associated with more than one white matter tract. The association between a locus and multiple phenotypes suggests that the locus has a broad genetic effect across multiple white matter tracts and may play an important role in the genetic underpinning of white matter microstructure.

Supervariants are identified for all 21 white matter tracts. The number of identified supervariants for each tract ranges from one to 28 (Fig. 2). The physical location of tracts in brain is displayed in Figure 3B. Several white matter tracts are associated with multiple supervariants. For example, for the anterior corona radiata, splenium of corpus callosum, genu of corpus callosum (GCC), and average FA, more than 20 supervariants are identified, respectively, which spread across a wide range of genomic regions, indicating the microstructure of white matter tract can be regulated by multiple genetic compartments across the whole genome.

**Figure 4.**
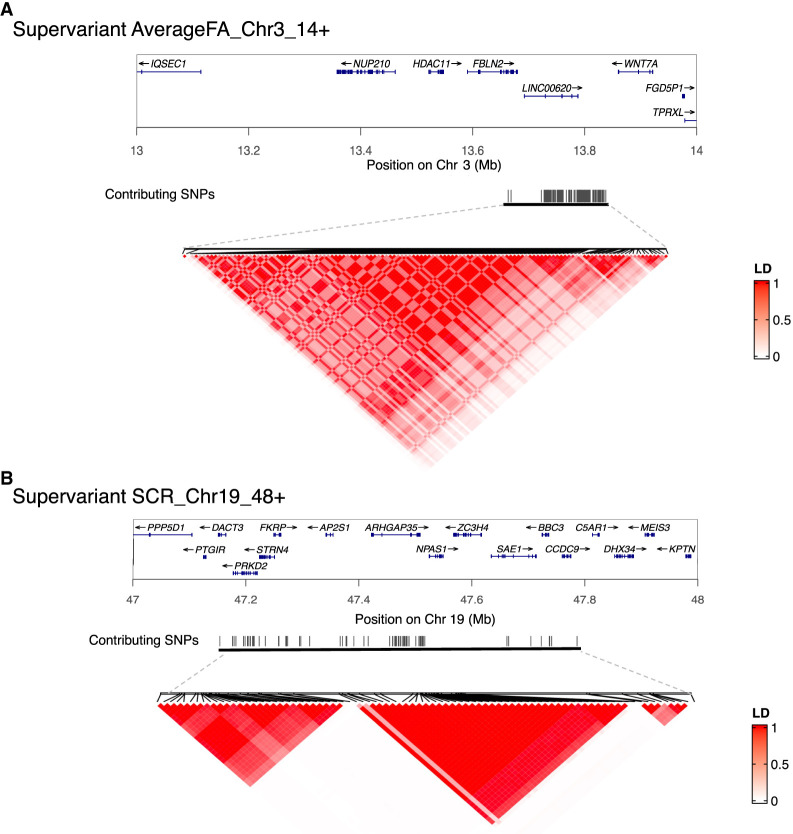
Selected supervariants and linkage disequilibrium structure. (*A*) Supervariant AverageFA_Chr3_14+. (*B*) Supervariant SCR_Chr19_48+. Black lines represent the physical location of selected SNPs on the chromosome. The color scale represents the linkage disequilibrium (*r*^2^) between each pair of selected SNPs.

In terms of the SNPs contributing to 314 supervariants, 19,798 unique SNPs are selected to construct these supervariants more than three times out of 10 replications. All contributing SNPs are detailed in Supplemental Table S2. SNPs selected to form one supervariant can be in one linkage disequilibrium (LD) block or multiple LD blocks. We show two example supervariants in Figure 4. Supervariant AverageFA_Chr3_14+ is constructed by several SNPs within one LD block (Fig. 4A), and supervariant SCR_Chr19_48+ is constructed by multiple SNPs within three LD blocks (Fig. 4B). On average, one supervariant contains 101.1 SNPs. Among all contributing SNPs, multiple SNPs are selected by more than one supervariant. For example, 31 SNPs are selected by 19 supervariants, and all of them locate in the SNP set Chr5_83, implying the genetic effect of this locus on the white matter microstructure.

We initially define SNP sets by extracting SNPs within a 1-Mbp window (Fig. 1A) for computational convenience and systematically perform genome-wide association tests. Beyond that, SNP sets can be defined by SNPs in genes that are not physically connected but have biological support in the literature or existing database. For example, we define a SNP set by SNPs located in nine genes (*SHANK2*, *LDLRAP1*, *NEFM*, *NEFH*, *NEFL*, *CLDN11*, *NRP1*, *INA*, *DLGAP2*) in the Gene Ontology (GO) gene set GO_NEUROFILAMENT (GO:0005883). Then, we perform supervariant identification and validation on the UKB British data set for this SNP set. The constructed supervariant with positive effect on the FA value of white matter tract FXST achieves *P* = 7.27 × 10^−10^ on the first part of data set and *P* = 1.88 × 10^−3^ on the second part. SNPs contributing to this supervariant are located in genes *NEFM*, *NEFL*, *DLGAP2*, and *NRP1*. These results suggest the genetic effect of this gene set on the white matter microstructure.

### External validation of supervariants

We validate the 314 supervariants in three independent validation data sets with European ancestry, including the UKBW (*n* = 1927), ABCD European (*n* = 4399), and HCP European (*n* = 319), and perform a meta-analysis (Fig. 1B). SNPs contributing to the supervariants equal to or more than three times out of 10 are extracted and aggregated into supervariants using additive coding on each external validation data set. Then, the associations between supervariants and phenotypes are assessed by a linear regression adjusting for covariates. Finally, the meta-analysis for the three validation data sets (*n* = 6645) is performed. The replication results are summarized in Supplemental Table S3. In the meta-analysis, 40 (12.7%) out of 314 identified supervariants pass the 1.6 × 10^−4^ (0.05/314) Bonferroni significance level. It is also noteworthy that 128 (40.7%) supervariants have *P*-values below the 0.05 level. All the 128 supervariants have concordant effect directions on the discovery and validation data sets. These results show a high degree of generalizability of findings among the European-ancestry cohorts. We find that all of 33 supervariants located in the SNP set Chr5_83 have *P*-values below the 0.05 level in the meta-analysis, suggesting consistent signals in this locus across independent data sets.

### Comparison with previous GWASs on white matter phenotypes

We compare the supervariant results with the previous largest GWASs for DTI-derived phenotypes (FA, MD, AD, RD, and MA) along 21 white matter tracts (Zhao et al. 2021a). First, we find that 204 out of 314 (65.0%) associations identified in the current study overlap with previous GWAS findings. Therefore, most of the identified associations are concordant with the previous GWASs on white matter phenotypes (Zhao et al. 2021a). Moreover, we replicate 78 out of the 151 genomic regions discovered by Zhao et al. (2021a) and identify additional 23 regions (Fig. 3A). Within 23 novel regions with potential effects on white matter microstructure, 31 supervariants are identified to be associated with the mean FA of 14 white matter tracts (Supplemental Table S4). It is worth mentioning that, among them, supervariant FXST_Chr8_25+ (validation *P* = 1.30 × 10^−4^) can be replicated with *P*-values lower than 1.6 × 10^−4^ (0.05/314) in the meta-analysis. In addition, supervariants GCC_Chr5_172+ (validation *P* = 3.9 × 10^−3^) and SCR_Chr19_48+ (validation *P* = 3.29 × 10^−2^) have *P*-values lower than 0.05. The effect directions of these three supervariants are consistent on the discovery set and three validation sets. These three supervariants preserve low *P*-values when further adjusting for the effect of SNPs identified in previous GWASs for DTI-derived phenotypes (Zhao et al. 2021a) in a conditional analysis, suggesting they are independent from previous identified loci (Supplemental Note S2).

### The shared genetic loci with complex traits and disorders

We conduct association lookups for contributing SNPs to 314 supervariants and SNPs within LD (*r*^2^ ≥ 0.6) to evaluate the shared genetic influences between white matter microstructure and other complex traits. In the NHGRI-EBI GWAS catalog (Buniello et al. 2019), the selected SNPs have been associated with a wide range of complex traits in different trait domains, such as brain structural traits (e.g., cortical volume and thickness), neurodegenerative diseases (e.g., Alzheimer's disease and Parkinson's disease), psychiatric disorders (e.g., bipolar disorder and schizophrenia), psychological traits (e.g., neuroticism), cognitive performance (e.g., intelligence and math ability), smoking, educational attainment, and anthropometric traits. These results are summarized in Supplemental Table S5. We highlight the colocalizations of SNPs contributing to three supervariants located in novel loci and with *P*-values lower than 0.05 in the meta-analysis of external validation data sets.

**Figure 5.**
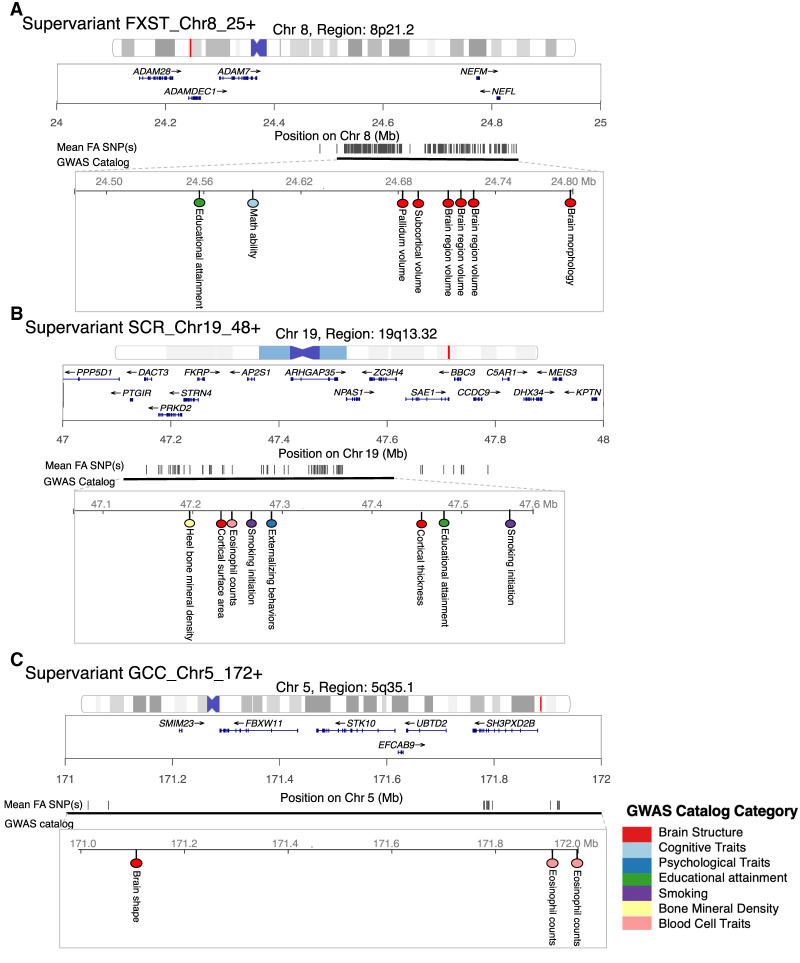
Selected supervariants with shared genetic loci associated with other complex traits and disorders. (*A*) Supervariant FXST_Chr8_25+. (*B*) Supervariant SCR_Chr19_48+. (*C*) Supervariant GCC_Chr5_172+. Black lines represent the physical location of selected SNPs on the chromosome. Physical location of SNPs that have been associated with other complex traits in the NHGRI-EBI GWAS catalog are shown. Colors of dots represent different trait categories.

Supervariant FXST_Chr8_25+ is composed of 344 SNPs in genomic region 8p21.2. These SNPs locate in genes *NEFM* and *NEFL* and intergenic regions and have been related to brain structural traits (e.g., pallidum volume [Zhao et al. 2019] and brain morphology [van der Meer et al. 2020]), math ability (Lee et al. 2018), and educational attainment (Fig. 5A; Okbay et al. 2022). Fornix-stria terminalis (FXST) connects the hippocampus and amygdala to the hypothalamus. Same as these brain structures, FXST is a critical component of the limbic system. Studies have shown that FXST is closely involved in emotion processing and memory (Douet and Chang 2015; Dzafic et al. 2019). In addition, the limbic system interacts with the basal ganglia, where pallidum is located. Of note, the basal ganglia and the limbic system have been associated with mathematical calculation and quantitative concepts in previous neuroimaging studies (Arsalidou and Taylor 2011; Pina et al. 2022). Our findings of the shared genetic loci may explain the connection between the limbic system, basal ganglia, and math ability.

Supervariant SCR_Chr19_48+ consists of 82 SNPs involving genes *ARHGAP35*, *SAE1*, *FKRP*, *STRN4*, *PRKD2*, and *DACT3* within genomic region 19q13.32. The contributing SNPs have been related to brain structural traits (e.g. cortical thickness [van der Meer et al. 2021] and surface area [Shadrin et al. 2021]), educational attainment (Okbay et al. 2022), smoking initiation (Justice et al. 2017; Liu et al. 2019), externalizing behaviors of attention deficit hyperactivity disorder (ADHD), substance abuse, and antisocial behavior (Fig. 5B; Karlsson Linner et al. 2021). The genomic region 19q13.32 where gene *APOE* is located has also been related to Alzheimer's disease (Moreno-Grau et al. 2019) and schizophrenia (Goes et al. 2015). Superior corona radiata (SCR) plays the role of transferring information to and from the cerebral cortex, where the disruption of white matter integrity has been found in patients with ADHD (Onnink et al. 2015), Alzheimer's disease (Yin et al. 2015), and schizophrenia (Meng et al. 2019) compared with healthy controls. Our findings suggest the shared genetic influence between SCR and multiple disorders.

Supervariant GCC_Chr5_172+ selects 17 SNPs located in gene *SH3PXD2B* within genomic region 5q35.1. Contributing SNPs have been associated with cortical surface morphology (Naqvi et al. 2021) and eosinophil count (Fig. 5C; Kichaev et al. 2019). GCC is the front part of corpus callosum, connecting the lateral and medial surfaces of the frontal lobes (Standring 2015). Corpus callosum serves as a hub between hemispheres and enables communications between two sides of our brain. Our findings suggest the shared genetic influence between white matter microstructure and brain structural traits. Overall, white matter microstructure has genetic links with a wide range of complex traits and diseases. Integrating the genetic findings with these traits and diseases may help explain the underlying mechanisms that lead to changes in brain structure and function and the risk of brain-related disorders.

### Biological annotations and gene-level analyses

We annotate 19,798 SNPs selected to form 314 identified supervariants using ANNOVAR (Wang et al. 2010) and summarize the function of SNPs and their corresponding genes in Supplemental Table S2. Regarding the physical positions of SNPs, 10,230 SNPs locate in 619 protein-coding genes, and the remaining are in the noncoding RNA genes or intergenic regions. Out of 306 SNPs within exon, 114 are nonsynonymous variants, and six SNPs are loss-of-function variants. Based on the criteria of SIFT score (Kumar et al. 2009) and PolyPhen-2 score (Adzhubei et al. 2013), seven nonsynonymous variants are predicted to be deleterious variants (Supplemental Table S6).

**Figure 6.**
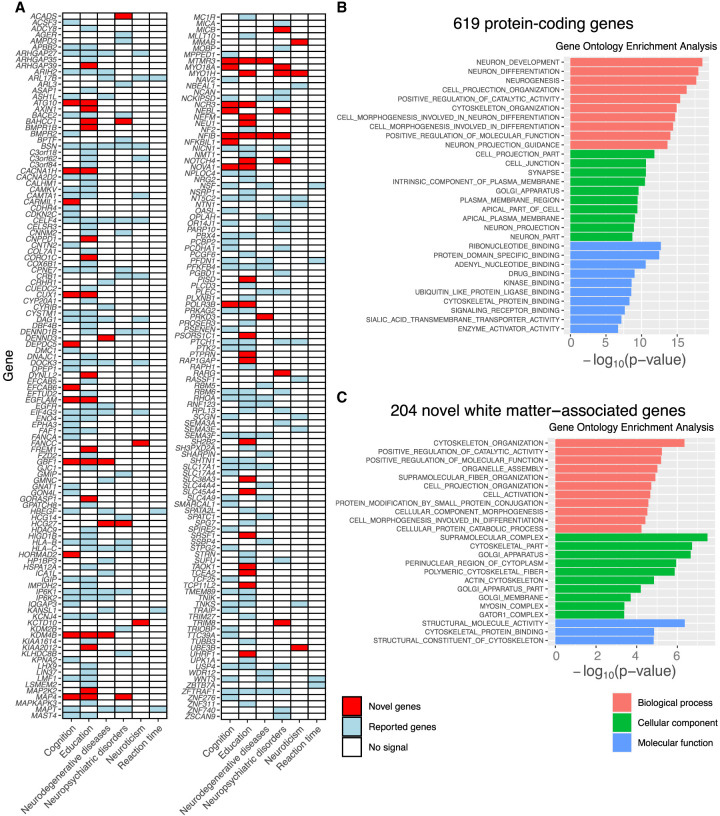
Gene-level analysis of 619 protein-coding genes. (*A*) Detected genes that have been linked to cognitive traits and brain-related disorders in previous GWASs. The novel and previously reported genes of human white matter are labeled with two different colors (red and blue, respectively). (*B*) Gene Ontology (GO) enrichment analysis for 619 protein-coding genes. At a FDR 5% level, the GO terms of 544 biological processes, 100 molecular functions, and 92 cellular components are significant. The top 10 of each category are shown. (*C*) GO enrichment analysis for 204 novel white matter–associated genes. At a FDR 5% level, the GO terms of 11 biological processes, three molecular functions, and 10 cellular components are significant.

For the detected protein-coding genes, we perform lookups in the NHGRI-EBI GWAS catalog (Buniello et al. 2019) and previous GWASs for white matter to explore their previously reported gene-trait associations. Our results replicate 415 genes reported by Zhao et al. (2021a) and some other genes reported in previous studies for human white matter (Sprooten et al. 2013, 2014; Verhaaren et al. 2015; Jian et al. 2018; Rutten-Jacobs et al. 2018; Zhao et al. 2021b; Zhang et al. 2021) and find 204 novel genes (Supplemental Table S7). Of the 619 detected genes, 227 have previously been implicated in cognitive function, education, neuroticism, neuropsychiatric disorders, neurodegenerative diseases, and reaction time, such as *ARIH2* (Lee et al. 2018; Kulminski et al. 2022) and *PTCH1* (Nagel et al. 2018; Thorp et al. 2021; Okbay et al. 2022). In particular, 57 out of the 227 pleiotropic genes are novel genes of white matter microstructure, and these findings substantially uncovered the gene-level pleiotropy between white matter microstructure and these traits (Fig. 6A).

To explore the biological interpretation of results, we conduct GO enrichment analysis (Liberzon et al. 2011) for 619 identified protein-coding genes and 204 novel white matter–associated genes, respectively. The results are shown in Figure 6, B and C, and Supplemental Tables S8 and S9. At a FDR 5% level, the GO terms of 544 biological processes, 100 molecular functions, and 92 cellular components are significant in the enrichment analysis for 619 protein-coding genes. Most of them are related to the development and regulation of the nervous system, such as neuron development (GO:0048666) and neuron differentiation (GO:0030182). As for the 204 novel white matter–associated genes, the GO terms of 11 biological processes, three molecular functions, and 10 cellular components are significant at a FDR 5% level, including cytoskeleton organization (GO:0007010) and positive regulation of catalytic activity (GO:0043085). We also perform the enrichment analysis of tissue-specific differentially expressed genes (DEGs) in 13 brain tissues (GTEx v8). We observe the enrichment of detected genes in the DEG of all brain tissues (*P* < 1.5 × 10^−9^ for 619 protein-coding genes and *P* < 1.1 × 10^−3^ for 204 novel white matter–associated genes), especially in the amygdala, putamen basal ganglia, and hypothalamus (Supplemental Table S10).

**Figure 7.**
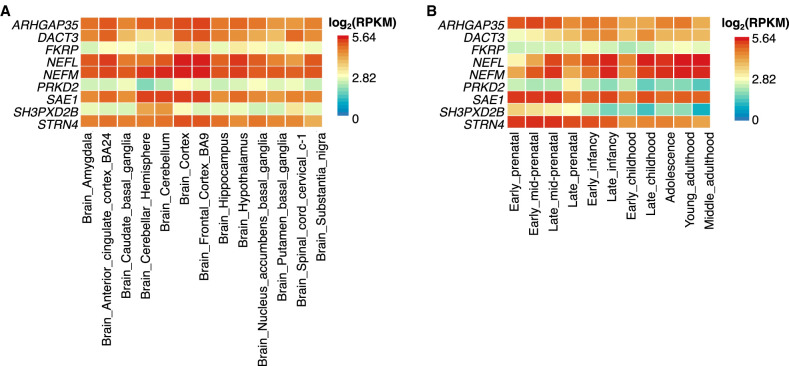
Expression of genes involved in three supervariants located in newly identified loci. (*A*) Expression of selected genes in 13 brain tissues in GTEx v8 database. (*B*) Expression of selected genes in 11 brain developmental stages in BrainSpan database.

We further examine the gene expression level of detected genes in brain tissues using the GTEx v8 (Lonsdale et al. 2013) and BrainSpan (2011) databases. We present the results of genes involved in three supervariants located in novel loci and with *P*-values lower than 0.05 in the meta-analysis of external validation data sets (Fig. 7). Genes *NEFM* and *NEFL* show high expression in all the brain tissues, suggesting their important role in brain structure or function. *ARHGAP35*, *SAE1*, and *STRN4* show moderate to high expression in all the brain tissues. The expression of *SH3PXD2B* is regional specific and concentrated in the cerebellum and cerebellar hemisphere (Fig. 7A). Regarding the development stage of brain samples in BrainSpan, *NEFM* and *NEFL* have higher expression from late childhood to adulthood, and *SAE1*, *STRN4*, and *ARHGAP35* show more expression from parental stage to infancy compared with adulthood (Fig. 7B).

## Discussion

In this study, we perform supervariant identification and validation to identify genetic loci associated with human white matter microstructure. We adopt the concept of the supervariant to account for potential multi-SNP effects. We discover and validate 314 supervariants in UKB White British. The results are further validated in three independent validation data sets. The identified 23 loci have not been reported in the previous GWASs on white matter microstructure (Zhao et al. 2021a). The identified loci share genetic influences with a wide range of complex traits. We annotate SNPs contributing to identified supervariants and perform GO enrichment analysis of corresponding genes. These genes are enriched in the development and regulation of the nervous system and DEG sets of brain tissues.

Comparing the results of supervariants and the previous largest GWASs on white matter phenotypes (Zhao et al. 2021a), we find that approximately two-thirds of the locus-trait associations identified in the current study are concordant with GWASs. Additionally, we identify 31 supervariants whose loci have not been reported in previous GWASs.

We report three supervariants located in novel loci and with *P*-values lower than 0.05 in the meta-analysis of external validation data sets. The contributing SNPs of supervariant FXST_Chr8_25+ locate in genes *NEFM* and *NEFL.* They both show exclusive expression in the brain tissues (Fagerberg et al. 2014). *NEFM* encodes the neurofilament medium chain, and *NEFL* encodes the neurofilament light chain. Neurofilaments are essential structural scaffolding proteins of neurons, which play a role in intracellular transport to axons and dendrites and are commonly used as a biomarker of neuronal damage (Khalil et al. 2018). Several studies have found the associations between protein NEFL level and Alzheimer's disease diagnosis and progression (Olsson et al. 2016; Zetterberg et al. 2016; Preische et al. 2019). The association between gene *NEFM* and Parkinson's disease has also been reported (Krüger et al. 2003).

Supervariant SCR_19_48+ involves the genes *ARHGAP35*, *SAE1*, *FKRP*, *STRN4*, *PRKD2*, and *DACT3*. Among them, *ARHGAP35*, *SAE1*, and *STRN4* show moderate to high expression in all the brain tissues. ARHGAP35, a RHO GTPase-activating protein in the radial glia-like neural stem cells within the ventricular zone of the medial ganglionic eminence, regulates dendritic spine formation, axon elongation, and pontine midline crossing (Kaur et al. 2020). SAE1-regulating protein structure and intracellular localization has been shown to promote human glioma progression (Yang et al. 2019). STRN4 belongs to the striatin family of scaffold proteins. The local expression of the gene *STRN4* in neuronal dendrites controls the dendritic spine morphology (Lin et al. 2017).

The contributing SNPs of GCC_Chr5_172+ are mainly mapped to the gene *SH3PXD2B*. This gene encodes an adapter protein required for podosome formation and is involved in cell adhesion and migration of numerous cell types (Mehes et al. 2019). The adapter protein encoded by *SH3PXD2B* belongs to the same family as the gene *SH3PXD2A*, which has been related to white matter hyperintensity (Persyn et al. 2020) and integrity (Standring 2015). The above evidence from existing literature supports our findings that these genes have potential genetic effects on white matter microstructure.

In addition to the three loci with *P*-values lower than 0.05 in the meta-analysis of external validation data sets, other novel loci are worth further analysis. Particularly, we identify 23 genomic regions that have not been associated with white matter phenotypes in the previous GWASs (Zhao et al. 2021a). We also detect 204 novel genes that have not been linked to white matter. These genes are enriched in biological processes, including cytoskeleton organization and positive regulation of catalytic activity. These genes could provide further research directions to understand the genetic architecture of white matter microstructure.

It is worth noting that supervariants can group SNPs in multiple loci together. For example, the supervariant SCR_Chr19_48+ is formed by SNPs within three LD blocks involving multiple genes. Thus, several potential genetic loci can be detected at the same time, which may also indicate the existence of joint effects among those genes. Such joint effects may have implications for the underlying mechanisms involving multiple genes.

Furthermore, our results indicate that the SNP set Chr5_83 may play an important role in the genetic underpinning of white matter microstructure. We identify 33 supervariants associated with multiple white matter tracts in this locus. More than half of the contributing SNPs in this locus locate in the gene *VCAN*, which is involved in cell adhesion, proliferation, migration, and angiogenesis. This gene shows a higher expression level in white matter than in other brain tissues (Thul and Lindskog 2018) and has been associated with white matter integrity (Elliott et al. 2018; Rutten-Jacobs et al. 2018).

There are multiple limitations and future directions to our study and analysis results. First, although we conduct both internal and external validation to increase the producibility, the replication rate on three independent validation data sets can be improved. It is worth mentioning that participants from the three data sets have different age ranges (middle and elderly ages for UKB, children and adolescents for ABCD, and young and middle ages for HCP). The heterogeneity in the age periods may influence the generalization of results. Second, we identify 314 supervariants on the discovery set and then apply 0.05/314 as the threshold for significance on the validation set. Further validation of the identified genetic loci in other independent data sets is needed. Third, we currently focus on the population with European ancestry. Replicating the identified loci in independent populations from other resources or ethnic groups would be important. Fourth, we initially define a SNP set by extracting SNPs within a 1-Mbp window for computational convenience as a systematic approach, so the SNPs contributing to a supervariant are constrained in a local region. This can be followed by considering predefined SNP sets such as genes within a pathway or gene set, which can be anywhere in the genome. Then, the corresponding supervariants can select SNPs located in functional-related genes on different chromosomes. Fifth, functional annotations of SNPs, such as annotation PCs (Li et al. 2020) and CADD (Kircher et al. 2014), provide rich biological information. We could further consider prioritizing SNPs based on annotation information when ranking the SNPs within the SNP set. For example, we can rank the SNPs based on their CADD scores instead of marginal *t*-statistic and then select the top SNPs to form supervariants following the same supervariants construction procedure. If multiple annotation scores are considered, we can perform SNPs ranking and select top SNPs based on each annotation score first separately. Then, the final supervariant is composed of SNPs that are selected from any of individual annotation score. In further investigation, we can evaluate the performance of different strategies to incorporate functional annotations. Sixth, we evenly divide the UKB White British data for discovery and internal validation of supervariant. However, different splitting ratios may impact the results of the analysis. After considering a variety of ratios for the two random subsets of the data set from extremely unbalanced 1:9, 2:8, 8:2, and 9:1 to relatively balanced 3:7, 4:6, 5:5, 6:4, and 7:3, we find that relatively balanced splitting ratios lead to robust results (results are detailed in the Supplemental Note S3). Thus, we choose to evenly divide the data set in the analysis. Additionally, we replicate our procedure 10 times, partly to limit the impact of the random splitting. Seventh, we use additive coding to aggregate SNPs selected to form supervariants, which may have limited power to identify epistatic or interactive effects among SNPs. However, supervariants can also be constructed using depth importance score from a forest-based model as the importance measure and indicator coding to aggregate signals (Hu et al. 2020; Hu et al. 2021). Simulation studies have shown that supervariants constructed in this way can detect SNPs with interactive effects (Hu et al. 2020). In further analysis, we can consider using indicator coding and depth importance score to detect potential epistatic effects among SNPs. Last but not least, the current study is limited to tract-specific FA parameters. Other parameters derived from DTI, such as MD, AD, RD, and MA, could provide complementary information and are worth further investigation to explore the genetic architecture underlying white matter microstructure.

## Methods

### Imaging phenotypes derived from DTI data

The DTI data used in this study come from the UKB, ABCD, and HCP studies. They are publicly available with the permission of the UKB (https://www.ukbiobank.ac.uk), ABCD study (https://nda.nih.gov/abcd/), and HCP (https://www.humanconnectome.org/software/connectomedb and https://www.ncbi.nlm.nih.gov/projects/gap/cgi-bin/study.cgi?study_id=phs001364.v1.p1). The data resources have obtained informed consent from all participants and have obtained approval from their research ethics committees or institutional review boards. The UKB study has ethics approval from the North West Multicentre Research Ethic Committee (approval number 21/NW/0157). All procedures in the ABCD study were approved by the centralized and institutional review boards at each data collection site (approval numbers 201708123 and 160091). All experimental procedures in the HCP study were approved by the institutional review boards at Washington University (approval number 201204036).

Detailed acquisition and preprocessing procedures have been described in brain imaging documentation (https://biobank.ctsu.ox.ac.uk/crystal/crystal/docs/brain_mri.pdf) for the UKB, Casey et al. (2018) for the ABCD, and Sotiropoulos et al. (2013) for the HCP. Standard registration and quality control are conducted for three data sets by the ENIGMA-DTI pipeline (Jahanshad et al. 2013; Kochunov et al. 2014). The workflows of processing and derivation of mean FA of white matter tracts are detailed by Zhao et al. (2021a) and in Supplemental Note S4. The ID and full names of these 21 white matter tracts are listed in Supplemental Table S11.

### Genotyping and quality control

We analyze the imputed genotype data from the UKB (Field ID: 22828), ABCD (Release 3.0), and HCP. In this study, we consider the biallelic variants and exclude SNPs with duplicated names and positions. We perform standard genetic quality controls on participants with both imaging and genotype data in each data set using PLINK (Purcell et al. 2007). Participants with missing genotype rates >10% are removed. We also exclude subjects whose genetic gender is inconsistent with self-reported gender and relatives closer than or equal to a third-degree relative (Bycroft et al. 2018). Genetic variants with low call rates (missing rate ≥ 10%), low minor allele frequency (minor allele frequency ≤ 0.01), disrupted Hardy–Weinberg equilibrium (*P*-value < 1 × 10^−7^), and low imputation quality (imputation INFO score < 0.8) are excluded. The positions of imputed genotypes from the ABCD are in the GRCh38 coordinate. We convert the positions to the GRCh37 coordinate by liftOver (https://genome.ucsc.edu/cgi-bin/hgLiftOver) in line with the UKB and HCP.

### Supervariant identification and internal validation on the discovery set

We use the data of the UKB participants with White British ancestry as the discovery set to limit the potential effect of population stratification. The ancestry assignment is based on the self-reported ethnic background (data-field 21000), whose accuracy was verified by Bycroft et al. (2018). After quality controls, the discovery set contains 30,842 participants and 8,900,385 SNPs.

To construct supervariants associated with phenotypes, a ranking and aggregation method (Song and Zhang 2014; Hu et al. 2020) is used, which is an adaptive method consisting of four steps.

Four steps are shown in Figure 1A and described in detail below. In step a, chromosomes are divided into nonoverlapping local SNP sets. We divide the whole genome into 2723 nonoverlapping local SNP sets according to the physical position so that each set consists of SNPs within a segment of physical length 1 Mbp. Each SNP set corresponds to a genomic region on chromosomes. We use the chromosome number and set number to denote each predefined SNP set. For instance, SNP set Chr1_1 consists of SNPs on Chromosome 1 with a base-pair position value falling between one and 999,999, and Chr1_2 composes of those with a base-pair position value between 1,000,000 and 1,999,999. In addition to defining SNP sets by extracting SNPs within a 1-Mbp window, SNP sets can also be defined by SNPs in genes that are not physically connected but have biological support in the literature or existing database. In step b, within each predefined SNP set, variants are ranked and reordered based on their positive and negative effect sizes on the phenotype, respectively, which leads to a ranking of SNPs in terms of their marginal contribution to the phenotype. Because the true effect sizes are unknown, we estimate the marginal effect of each SNP on the phenotype using a linear regression model while controlling for covariates: age (at imaging), sex, image site, age-squared, the interaction between age and sex, the interaction between age-squared and sex, and the top 10 PCs provided by the UKB. We use the *t*-test statistic for testing whether the coefficient of variant is significantly different from zero to order SNPs both descendingly and ascendingly so that variants with positive or negative effects are accounted for (Song and Zhang 2014). In step c, we empirically determine the number of top SNPs to form a supervariant following the method of Hu et al. (2020). Specifically, we explore each possible cutoff value, aggregate the top SNPs using additive coding, and test the association between the aggregated score with the phenotype while adjusting for covariates. Then, we select the best cutoff value that achieves the lowest *P*-value in the association test. Because the *t*-test statistics of SNPs are ranked both descendingly and ascendingly, two supervariants with the positive effect and negative effect are formed, respectively, for each predefined SNP set. In step d, top SNPs selected in step c within each SNP set are aggregated using additive coding (summation of the number of minor alleles of each SNP) into supervariants. A supervariant is denoted by the phenotype, SNP set, and effect direction as “pheno_set_+/−”. We test the association between aggregated score and phenotype while adjusting for age (at imaging), sex, image site, age-squared, the interaction between age and sex, the interaction between age-squared and sex, and the top 10 PCs.

We consider the following discovery and internal validation procedure (Fig. 1B; Hu et al. 2021). The complete set is randomly divided into two sets with equal sizes (*n* = 15,421 for each set): one for the construction of supervariants and the other for validation. The minor allele of each SNP is kept the same on two parts of data set. We apply the aforementioned ranking and aggregation method for supervariant construction on the first part of the data set. After the construction of the supervariants, we then validate their associations with the white matter phenotype through linear regression on the second part. We control for age (at imaging), sex, image site, age-squared, the interaction between age and sex, the interaction between age-squared and sex, and the top 10 PCs in the regression analyses as covariates to remove potential bias. We use 4.17 × 10^−7^ (i.e., 0.05/(2723 × 2 × 22)) as the threshold for supervariant candidacy on the first part of data set because 5446 supervariants and 22 phenotypes are considered. A supervariant is regarded as validated if its linear regression coefficient achieves the level of 0.05/22 significance on the second part of the data set, adjusting for the number of phenotypes. We repeat the above procedure 10 times and retain the supervariants that can be discovered and validated multiple times.

### External validation of supervariants

We replicate the supervariants on three validation data sets consisting of participants with European ancestry: the UKBW (n = 1927), ABCD European (n = 4399), and HCP European (n = 319). The ancestry assignment in the ABCD and HCP are based on self-reported ethnic groups. The relatedness between participants is checked based family IDs. We random select one participant for each family ID to remove relatedness. SNPs contributing to the supervariants (equal or more than three times out of 10 times replication) are extracted. SNPs with inconsistent minor alleles to the discovery sets are flipped. Then, contributing SNPs are aggregated into supervariants using additive coding on each external validation data set. A linear regression on FA phenotypes is used to test the significance of association with supervariants controlling for age, sex, age-squared, the interaction between age and sex, the interaction between age-squared and sex, and the top 10 PCs properly. After obtaining *P*-values for each supervariant on three data sets, a meta-analysis is performed on these validation data sets using METAL (Willer et al. 2010) with the sample-size weighted approach.

### Biological annotation and gene-level analyses

We annotate 19,798 SNPs selected to form 314 identified supervariants using ANNOVAR (Wang et al. 2010). Nonsynonymous variants are predicted to be deleterious when the SIFT score (Kumar et al. 2009) is lower than 0.05 and the PolyPhen-2 score (Adzhubei et al. 2013) is larger than 0.9. For 619 protein-coding genes, we perform the lookups of previously reported gene-trait associations with *P*-value <5 × 10^−8^ in the NHGRI-EBI GWAS catalog 2022-11-08 (Buniello et al. 2019). We focus on brain-related complex traits and characterize them into six groups: cognitive (e.g., general cognitive ability, cognitive performance, math ability, and intelligence), education (e.g., years of education and college completion), reaction time, neuroticism, neurodegenerative diseases (e.g., Alzheimer's disease, Parkinson's disease), and neuropsychiatric disorders (e.g., major depressive disorder, schizophrenia, bipolar disorder, ADHD, alcohol use disorder, and autism spectrum disorder). The GO enrichment analysis of identified genes is performed by GENE2FUNC in FUMA (Watanabe et al. 2017) based on the Molecular Signatures Database (MSigDB; version 7.0) (Liberzon et al. 2011). We also perform the enrichment analysis of tissue-specific DEGs in 54 tissues (GTEx v8) via GENE2FUNC in FUMA (Watanabe et al. 2017).

## Supplementary Material

Supplement 1

Supplement 2

Supplement 3

Supplement 4

Supplement 5

Supplement 6

Supplement 7

Supplement 8

Supplement 9

Supplement 10

Supplement 11

Supplement 12
