# Supplementary material for: Identification and validation of supervariants reveal novel loci associated with human white matter microstructure
Source: Genome Res. 2024 Jan;34(1):20–33. doi: 10.1101/gr.277905.123 (PMC10904010; doi:10.1101/gr.277905.123)
Supplement: Supplement 12 [file Supplemental_Materials.pdf]

Supplemental Materials for

Identification and validation of supervariants reveal novel loci associated with human white matter microstructure

The supplementary materials include:

Supplemental Notes

S1. Simulation studies for supervariant discovery and internal validation procedure.

S2. Conditional analysis of known common SNPs for supervariants.

S3. Analysis of the impact of different splitting strategies on the power of supervariant identification.

S4. Image processing and derivation of mean fractional anisotropy.

Supplemental Tables:

Table S1: 314 supervariants discovered and validated in at least 6 out of 10 times replication on the discovery set.

Table S2: Summary of SNPs contributing to 314 supervariants identified on the discovery set.

Table S3: Validation results of supervariants on three validation datasets and meta-analysis.

Table S4: 31 supervariants located in newly identified loci.

Table S5: Selected SNPs and tagged SNPs for supervariants that have previously been identified at $P < 5 \times 10^{-8}$ in GWAS of diseases and traits listed in the GWAS catalog 2022-11-08.

Table S6: Nonsynonymous variants predicted to be deleterious based on SIFT score and PolyPhen-2 score.

Table S7: Summary of novel genes associated with human white matter.

Table S8: Summary of Gene Ontology enrichment analysis of 619 protein-coding genes.

Table S9: Summary of Gene Ontology enrichment analysis of 204 protein-coding genes.

Table S10: Summary of enrichment analysis of tissue-specific differentially expressed gene set.

Table S11: ID and full name of 21 white matter tracts.

References

Supplementary Notes

S1. Simulation studies for supervariant discovery and internal validation procedure

To evaluate if the discovery and internal validation procedure can control false positives, we perform simulation studies to apply this procedure on 22 simulated null phenotypes. Specifically, we directly use the 2,723 SNP sets in the real data analysis as the genotype data. Then, we randomly generate 22 continuous phenotypes without genetic effects by $y_{ik} = x_{ik} + \epsilon_{ik}$, where covariate $x_{ik} \sim N(0,1), \epsilon_{ik} \sim N(0,1),\ k = 1,..,22$.

We follow the same discovery and internal validation procedure shown in Figure. 1B (threshold on part 1: 0.05/(22×2723×2), threshold on part 2: 0.05/22) and repeat our proposed procedure 10 times. We repeat this simulation 10 times to evaluate the type I error rate. At the thresholds mentioned above, no SNP sets pass both discovery and validation requirements more than 5 times, suggesting that this procedure used in real data analysis can well control type I error.

S2. Conditional analysis of known common SNPs for supervariants

To evaluate if novel loci identified in this study are independent from previous ones in GWAS. We perform a conditional analysis for the 539 unique leading SNPs identified in the previous largest GWAS on DTI-derived phenotypes (Zhao et al. 2021a). Specifically, we consider two strategies. First, we include one leading SNP as a covariate in the regression model of supervariants and phenotype while adjusting for age (at imaging), sex, image site, age-squared, the interaction between age and sex, the interaction between age-squared and sex, and top 10 PCs at one time. Second, we aggregate the 539 leading SNPs into one single score by additive coding and include it as a covariate into the regression model of supervariants and phenotype to adjust for the joint effects of 539 SNPs. We summarize the original *p*-values of three supervariants, the maximal *p*-values of supervariants among the conditional analysis of 539 known SNPs, and the *p*-values of supervariants after adjusting for 539 SNPs jointly in the following table. The supervariants preserve low *p*-values in the conditional analysis, suggesting these loci are independent from previous ones.

Supervariant	*P*-value without adjustment for known SNPs	Maximal *p*-value with adjustment for a known SNP among 539 conditional analyses	*P*-value with adjustment for 539 SNPs jointly
FXST_Chr8_25+	9.39e-14	1.77e-13	1.07e-13
GCC_Chr5_172+	1.28e-14	2.74e-14	1.34e-14
SCR_Chr19_48+	1.27e-15	2.18e-15	1.28e-15

S3. Analysis of the impact of different splitting strategies on the power of supervariant identification

To evaluate how the splitting strategy may impact the power of the analysis, we consider a variety of splitting ratios for the two random subsets of the dataset from extremely unbalanced 1:9, 2;8, 8:2, and 9:1 to relatively balanced 3:7, 4:6, 6:4, and 7:3. We investigate how robust the supervariants that we identify through the evenly splits versus these different splitting ratios.
Specifically, in the UKB British dataset, we randomly split the dataset with splitting ratios 1:9, 2:8, 3:7, 4:6, 6:4, 7:3, 8:2, and 9:1, respectively, and then follow the same steps as when we use the 5:5 ratio to construct and validate supervariants. In the following table, we summarize the number of supervariants that are reproducible from these splitting ratios and the percentage of overlaps with the use of the 5:5 splitting ratio.

Ratio	Number of reproducible supervariants	Percent of overlaps with the 5:5 ratio
1:9	12	30.0%
2:8	22	55.0%
3:7	32	80.0%
4:6	35	87.5%
6:4	38	95.0%
7:3	29	72.5%
8:2	24	60.0%
9:1	12	30.0%

Under ratios 6:4, 4:6, 3:7, and 7:3, most of the supervariants that we report can also be identified. However, as the sizes of the two parts become more unbalanced, the number of identified supervariant decreases. The small first part of the dataset may result in an inaccurate estimation of effect size and rank, while the small second part may lead to not enough sample size for the association test when validating the supervariants. Thus, the different splitting strategies affect the results of the analysis, but relatively balanced splitting ratios lead to robust results.

S4. Image processing and derivation of mean fractional anisotropy

We perform consistent standard registration and QC steps based on the ENIGMA-DTI pipeline (Jahanshad et al. 2013; Kochunov et al. 2014) for different datasets (http://enigma.ini.usc.edu/protocols/dti- protocols/). Specifically, we first use linear registration to register each of the FA images to the ENIGMA fractional anisotropy (FA) template at 1 × 1 × 1 mm spatial resolution on the MNI-ICBM-152 standard space. We then apply nonlinear registration to align the linearly registered FA images to this standard space and mask the registered FA images with the template brain mask. Next, we project the ENIGMA skeleton onto the registered images. Finally, we extract the tract-based tract-averaged mean for FA images. The full data analysis steps are summarized as follows.

1. The UKB brain imaging team carried out image preprocessing steps to process the dMRI data, removed problematic raw images, and conducted corrections for eddy currents, head motions, outlier slices, and gradient distortions. The individual images of FA were generated by fitting DTI models using the FSL software (https://fsl.fmrib.ox.ac.uk/fsl/fslwiki).
2. Following the ENIGMA-DTI pipeline, we examine the directional information of the primary eigenvector V1. Specifically, for each FA image, we generate the directional information and manually check whether the gradients in V1 aligned appropriately along with the FA image. Example images with good and bad quality can be found in (Zhao et al. 2021b).
3. For each FA image, we generate the FA brain mask. We zero the end slices and slightly erode the image (https://github.com/pnlbwh/TBSS/blob/master/docs/TUTORIAL.md). Abnormal values at the boundary of FA images are excluded.
4. We linearly register FA images to the ENIGMA FA template on the MNI-ICBM-152 space. We apply the 9-parameter linear registration and use the correlation ratio as cost function. We manually check the registration performance and remove the FA images with bad performance.
5. We nonlinearly register the linearly aligned FA images to the ENIGMA FA template. We manually check the registration performance and remove the FA images with bad performance.
6. We skeletonize the registered FA images by projecting the ENIGMA skeleton onto these images. More details can be found in (Smith et al. 2006).
7. We extract the regional mean of the skeletonized FA images within each of the predefined white matter tracts, according to the JHU ICBM-DTI-81 white matter atlas.

References

Jahanshad N, Kochunov PV, Sprooten E, Mandl RC, Nichols TE, Almasy L, Blangero J, Brouwer RM, Curran JE, de Zubicaray GI et al. 2013. Multi-site genetic analysis of diffusion images and voxelwise heritability analysis: a pilot project of the ENIGMA-DTI working group. *Neuroimage* **81**: 455-469.

Kochunov P, Jahanshad N, Sprooten E, Nichols TE, Mandl RC, Almasy L, Booth T, Brouwer RM, Curran JE, de Zubicaray GI et al. 2014. Multi-site study of additive genetic effects on fractional anisotropy of cerebral white matter: Comparing meta and megaanalytical approaches for data pooling. *Neuroimage* **95**: 136-150.

Smith SM, Jenkinson M, Johansen-Berg H, Rueckert D, Nichols TE, Mackay CE, Watkins KE, Ciccarelli O, Cader MZ, Matthews PM et al. 2006. Tract-based spatial statistics: voxelwise analysis of multi-subject diffusion data. *Neuroimage* **31**: 1487-1505.

Zhao B, Li T, Yang Y, Wang X, Luo T, Shan Y, Zhu Z, Xiong D, Hauberg ME, Bendl J. 2021a. Common genetic variation influencing human white matter microstructure. *Science* **372**.

Zhao B, Zhang J, Ibrahim JG, Luo T, Santelli RC, Li Y, Li T, Shan Y, Zhu Z, Zhou F et al. 2021b. Large-scale GWAS reveals genetic architecture of brain white matter microstructure and genetic overlap with cognitive and mental health traits (n = 17,706). *Mol Psychiatry* **26**: 3943-3955.
